# Pilot study on the effects of preservatives on corneal collagen parameters measured by small angle X-ray scattering analysis

**DOI:** 10.1186/s13104-021-05494-y

**Published:** 2021-02-27

**Authors:** Susyn Joan Kelly, Lizette duPlessis, John Soley, Frazer Noble, Hannah Carolyn Wells, Patrick John Kelly

**Affiliations:** 1 Department of Clinical Sciences, Ross University of Veterinary Medicine, Basseterre, Saint Kitts and Nevis; 2 School of Engineering and Advanced Technology, Massey University, Palmerston North, 4442 New Zealand; 3 Department of Anatomy and Physiology, Electron Microscope Unit, Faculty of Veterinary Science, University of Pretoria, Onderstepoort, 0110 South Africa

**Keywords:** Collagen, SAXS, Cornea, Formalin, Glutaraldehyde, TritonX

## Abstract

**Objective:**

Small angle X-ray scattering (SAXS) analysis is a sensitive way of determining the ultrastructure of collagen in tissues. Little is known about how parameters measured by SAXS are affected by preservatives commonly used to prevent autolysis. We determined the effects of formalin, glutaraldehyde, Triton X and saline on measurements of fibril diameter, fibril diameter distribution, and D-spacing of corneal collagen using SAXS analysis.

**Results:**

Compared to sections of sheep and cats’ corneas stored frozen as controls, those preserved in 5% glutaraldehyde and 10% formalin had significantly larger mean collagen fibril diameters, increased fibril diameter distribution and decreased D-spacing. Sections of corneas preserved in Triton X had significantly increased collagen fibril diameters and decreased fibril diameter distribution. Those preserved in 0.9% saline had significantly increased mean collagen fibril diameters and decreased diameter distributions. Subjectively, the corneas preserved in 5% glutaraldehyde and 10% formalin maintained their transparency but those in Triton X and 0.9% saline became opaque. Subjective morphological assessment of transmission electron microscope images of corneas supported the SAXS data. Workers using SAXS analysis to characterize collagen should be alerted to changes that can be introduced by common preservatives in which their samples may have been stored.

## Introduction

The protein collagen provides strength and structure to tissues including the cornea. The basic collagen molecule consists of a repeating series of three amino acids which coil together in a triple helix [1] These molecules align in a staggered side-by-side fashion forming collagen fibrils with D-spaces representing areas of high and lower collagen molecule overlap. The arrangement of collagen fibrils in tissues influences their physical properties: a mainly parallel arrangement imparts strength (tendon) while a more mesh-like structure provides flexibility and resistance to tear (skin) [2]. In the eye, collagen is the main structural component of the cornea providing strength and the precise curvature required for refraction. The arrangement of the fibers in the peripheral cornea and limbus help maintain corneal curvature when extraocular eye muscles impose stresses during eye movement [3, 4]. The collagen fibers in the cornea must have short-range ordering for corneal transparency. There is destructive interference of light scattered by the fibrils in all directions, except for forward which enables the cornea to be transparent and pass light to the retina [5].

Small angle X-ray scattering (SAXS) is a sensitive method for analyzing nanostructures of 1–400 nm. X-rays transiting a sample are diffracted by its components and the resulting scatter-patterns provide information on their shape and size. The process is non-destructive and requires minimal sample preparation [6]. Although SAXS has been used to analyze a wide range of biologicals, it has been particularly useful in studies of the cornea [7–13], and in describing eye lesions [14].

Although SAXS can be performed on unprocessed tissue, often samples have been fixed in preservatives to prevent autolysis and introduce rigidity necessary for tissue-sectioning. The changes to collagen seen with transmission electron microscopy (TEM) are well described [15] but there is minimal information on the effects of fixation on collagen parameters measured by SAXS. Interfibrillar-spacing in bovine corneas fixed in 2.5% glutaraldehyde was similar to that in fresh-corneas but D-spacing significantly decreased [16]. After 48-h in an unspecified formalin formulation, rat tendon had similar D-spacing to tendon stored in PBS [17]. Freezing of human corneas had no effect on X-ray scattering patterns [11].

Knowing how fixation modifies tissue ultrastructure is very important and processing methods need to be chosen carefully to preserve features of interest. To provide information on how collagen parameters determined by SAXS are affected by commonly used preservatives we studied treated and untreated sheep and cats’ corneas. TEM was performed to complement the SAXS data.

## Methods

### Samples

Clinically normal corneas were collected from two adult female sheep (1 year-old) immediately after slaughter at an abattoir and from an adult male and adult female cat necropsied at Massey University for reasons unrelated to the study. Only the central areas of the corneas, excluding the outer 2–4 mm [18], were used in the experiments as this has the most uniform collagen fibril arrangement in a variety of species including primates, cattle, horses, pigs, rabbits and mice [3, 18, 19]. For each animal, the central areas of both corneas were removed with a new scalpel blade and dissected to provide five approximately equally sized pieces. One piece was used as an untreated control. As freezing has no effect on X-ray scattering patterns [11], these control samples were immediately wrapped tightly in clingwrap to prevent dehydration and stored at − 80 °C. The remaining four pieces from each eye of each animal were placed in either 2 mL of 5% glutaraldehyde (G), 10% formalin (F), Triton X (T) or 0.9% saline (S) prepared by standard methods [20]. Each of the 4 cats’ and 4 sheep corneas thus provided a control untreated sample and samples treated with the four standard preservatives.

After four-days of preservation the samples were tested for transparency by subjectively observing a 4 × 4 mm cross (1-point black line) through the sample (see Additional file 1) and analyzed by SAXS. Immediately thereafter, samples were fixed in Karnovsky’s fixative for evaluation by TEM.

### SAXS

**Fig. 1:**
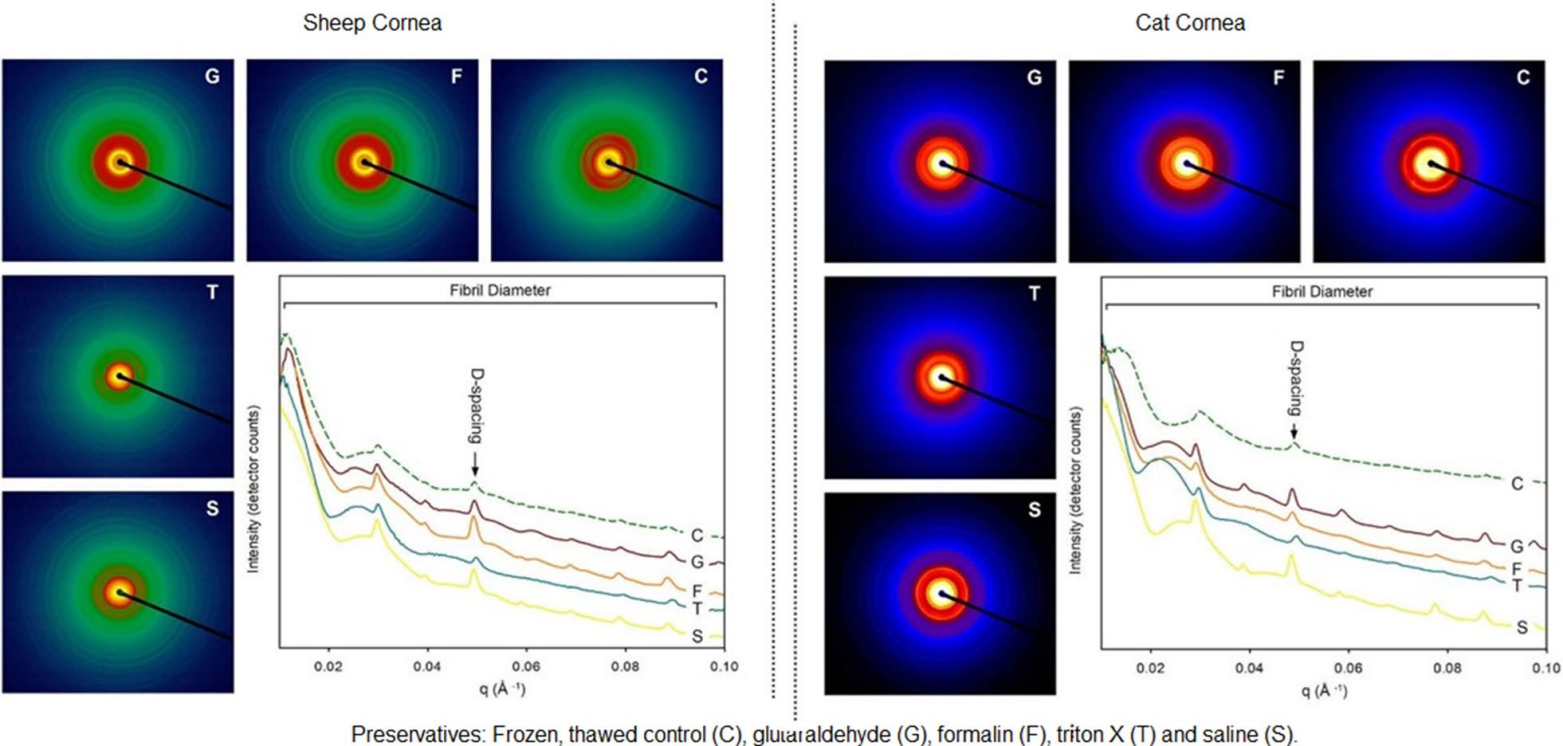
Photo images of 2D small angle X-ray scattering patterns produced by frozen and thawed control corneas and the preserved corneas. The graph shows the intensity profiles over the measured q-range for all the samples. Arrow indicates peak (0.045–0.055 Å^−1^) used to determine D-spacing and the full q-range (0.01–0.1 Å^−1^) for the fibril diameter

At the SAXS/WAXS beamline of the Australian Synchrotron frozen untreated control samples were thawed immediately before the experiment. Controls and preserved samples were mounted flat-on to the X-ray beam (optical axis from anterior to posterior). After excess preservative was gently removed from samples with a gauze swab, all samples were sealed in Kapton tape to prevent tissue dehydration and to hold the samples in place while surface diffraction measurements were made using a 3 × 3 grid with 0.25 mm spacing between points. A high-intensity undulator source from a cryo-cooled Si (111) double-crystal monochrometer was utilized with an energy resolution of 10^–4^. Beam size was 250 × 80 µm and total photon flux ~ 2 × 10^12^photons s^−1^ (acquisition rate 1–5 s). All diffraction patterns were calibrated with silver behenate and recorded at 12 keV using a Pilatus 1 M detector at 3337 mm. Data was processed with ScatterBrain software. D-spacing was calculated by comparing diffraction peak positions of the 5th order peak with the calibrant to determine q-values, after background subtraction, using Gaussian approximations (Fig. 1). Fibril diameters were determined over the full q-range (0.01–0.1 Å^−1^) (Fig. 1) by applying the ‘cylinder AR’ model using “Irena”, a macro developed for analyzing particle size distributions in SAXS data [21] running in a data analysis tool (Igor Pro, Wavemetrics).The variability of fibril diameters measured in each sample was determined from the full width half maximum of the normal distribution of the histogram plot of the frequency vs. fibril diameter.

### TEM

Corneas in Karnovsky’s fixative were trimmed, post-fixed in osmium tetroxide (0.1 M), dehydrated with ethanol washes, and embedded in epoxy resin (TAAB812, UK). Ultra-thin sections (70–90 nm) were cut (LeicaEMUC7, DE), mounted on copper-grids, stained with at 80 kV.

### Image processing

A Graphical User Interface (GUI), was used to measure the collagen fibrils in end-on TEM images. A pixel-to-nanometer scale was computed with the GUI and used to detect contours which, with Delaunay triangulation and Voronoi diagrams, enabled measurements of fibril diameters and distances to nearest neighbors.

## Results

### Transparency test

The printed cross was clearly visible through the control corneas (see Additional file 1) and those preserved in G and F. It was less clearly visible through corneas preserved in S and not visible through corneas stored in T.

### SAXS

Scatter patterns and their associated intensity vs. q-range plots are shown in Fig. 1 for the sheep and cats’ corneas treated with the various preservatives. The 5th order peak was used for measuring D-spacing and the full q-range (0.01–0.1 Å^−1^) for fibril diameter (Table 1). Relative to the controls, fibril diameters and distributions for both the sheep and cats’ corneas preserved in G were significantly higher (P < 0.05). However, they had significantly lower D-spacing than the controls (P < 0.05). Similarly, corneas preserved in F had fibril diameters and distributions significantly higher than the controls and significantly lower D-spacing (P < 0.05). The fibril diameters for corneas preserved in T were significantly greater (P < 0.05) than controls and the largest recorded. The fibril diameter distributions, however, were significantly lower than for the controls. The D-spacing was increased in both the cats’ and the sheep corneas but this was only significant in the latter (P < 0.05). Of all the preservatives, samples in S had values closest to those of controls with no significant differences between the D-spacing of the sheep and cats’ corneas. Compared to controls, however, fibril diameters in both species were significantly higher (P < 0.05) and diameter distributions significantly lower.

**Table 1:** Results of small angle X-ray scattering analysis to determine average (standard deviation) D-spacing and fibril diameter and results from the transmission electron microscopy image analysis to determine average (standard deviation) fibril diameter of sheep and cats’ corneas treated with 5% glutaraldehyde (G), 10% formalin (F), Triton X (T), and 0.9 % saline (S). *P*-values relate to a significance test between the various preservatives relative to the control untreated cornea sample values (C)

Species	Collagen properties	Preservative				Control
		**G**	**F**	**T**	**S**	**C**
Sheep	D-spacing (nm)	64.96 (0.09)	65.16 (0.04)	65.58 (0.02)	65.36 (0.04)	65.42 (0.01)
	*P*-value	< 0.05	< 0.05	< 0.05	0.06	–
	SAXS fibril diameter (nm)	37.08 (0.23)	36.44 (0.24)	38.27 (0.32)	36.68 (0.20)	35.52 (0.15)
	*P*-value	< 0.05	< 0.05	< 0.05	< 0.05	–
	Fibril diameter distribution (nm)	5.40 (0.45)	5.22 (0.20)	3.99 (0.34)	3.50 (0.36)	4.83 (0.31)
	*P-*value	< 0.05	< 0.05	< *0.05*	< 0.05	–
	TEM fibril diameter	35.88 (3.27)	38.35 (3.28)	52.37 (10.51)	40.81 (5.69)	32.10 (2.70)
	*P-*value	< 0.05	< 0.05	< 0.05	< 0.05	–
Cat	D-spacing (nm)	64.71 (0.06)	64.83 (0.06)	65.30 (0.11)	65.15 (0.01)	65.22 (0.01)
	*P-*value	< 0.05	< 0.05	0.29	0.07	–
	SAXS fibril diameter (nm)	40.50 (0.25)	39.18 (0.31)	41.73 (0.48)	38.50 (0.23)	37.26 (0.24)
	*P-*value	< 0.05	< 0.05	< 0.05	< 0.05	–
	Fibril diameter distribution (nm)	4.89 (0.28)	5.01 (0.21)	4.21 (0.37)	3.23 (0.37)	4.53 (0.12)
	*P-*value	< 0.05	< 0.05	< 0.05	< 0.05	–
	TEM fibril diameter	43.21 (5.12)	47.38 (5.74)	45.69 (7.65)	44.29 (6.43)	42.07 (4.13)
	*P-*value	< 0.05	< 0.05	< 0.05	< 0.05	–

### TEM

**Fig. 2:**
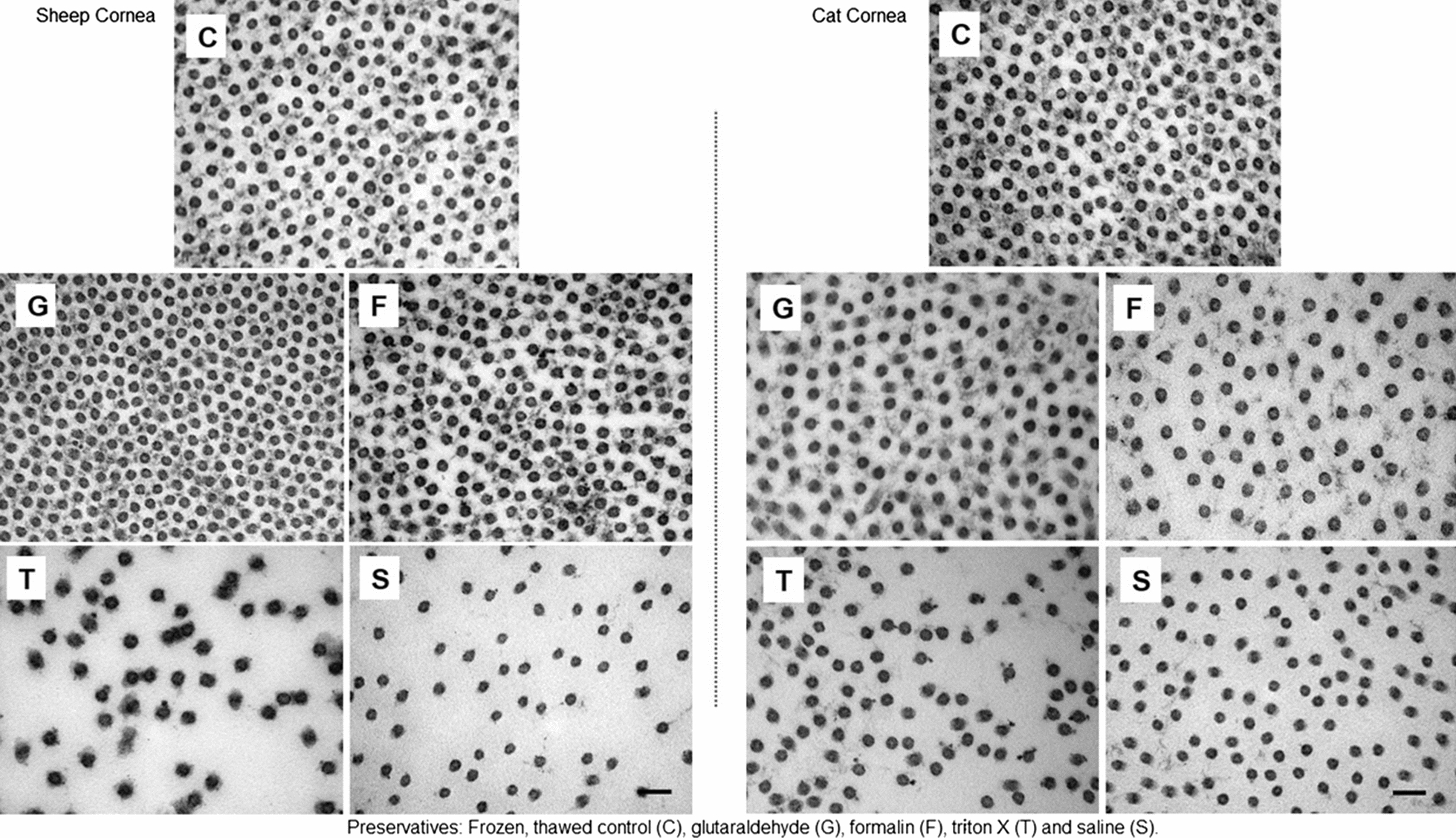
Transmission electron micrographs depicting collagen fibril cross-sections in the stroma of sheep (left) and cat (right) corneas freeze/thawed or preserved for 5 days followed by fixation in Karnovsky’s fixative and processing for TEM

It should be noted that the steps used in processing the samples for TEM affect the morphology of collagen [17] and it was not therefore possible to perform quantitative comparisons of results obtained by SAXS and TEM. However, subjective morphological assessment of the TEM images strongly supported the SAXS data showing, for example, that fibril diameters in the G, F samples were significantly smaller than the S and control samples while the T samples were significantly larger than the saline and control corneas (Fig. 2).

Additionally, visual inspection of Fig. 2 suggests significant variation in the interfibrillar spacing/distance, number of fibrils in a given area, and the amount of interfibrillar matrix between the T and saline samples with the controls, and, to a lesser-degree, the samples preserved in F and G. As with the SAXS analyses, fibril diameters were significantly larger in the T samples and significantly smaller in the saline samples compared to the control samples (Fig. 2). Epithelial and endothelial cells, when visible in the F and G preserved corneas, had normal morphology. No cells were seen in sections showing the anterior and posterior areas of corneas preserved in T and saline.

### TEM image processing

A pattern of fibril diameter distribution like that in the SAXS study was noted (Table 1). The diameters of the fibrils in both the sheep and cats’ normal controls were significantly smaller than those in the samples preserved in G, F, T and S.

## Discussion

While modern techniques enable detailed analyses of the nanostructure of biological materials, the processing required before analysis often leads to significant changes in the shape and size of tissue components. Although there is reasonable data on changes seen in TEM, caused by processing TEM [15, 16, 22, 23], there is little on the effects of tissue processing on parameters measured by SAXS. Our study shows commonly used preservatives introduce significant changes in collagen parameters measured with SAXS. Formalin and G significantly decreased D-spacing and increased collagen fibril diameters. Both F and G are relatively small molecules that readily penetrate collagen and form cross linkages that bind collagen molecules together and decrease D-spacing. Before such cross links can form, however, it has been suggested that the hypotonic fixative solution moves into the fibrils and causes them to swell and have increased fibril diameter [16]. This is consistent with our findings of increased fibril diameters in samples stored in F and G. With TEM, the fibrils in G and F samples appeared to have relatively uniform diameters with short-range order interfibrillar spacing. This is essential for optical transparency [2, 3] which was shown by the F and G samples in the transparency testing. Further, epithelial and endothelial cells in corneas preserved in G and F appeared normal, another requirement for corneal transparency. The presence of specialized water-soluble structural crystalline proteins and high levels of enzymes such as aldehyde-dehydrogenase and transketolase in the cytoplasm of epithelial cells [24] results in refractive indices of the cytoplasm and cell organelles within a range that does not produce scattering of light.

TritonX is a non-ionic detergent used to produce implantable acellular matrix scaffolds of heart valves [25], tendons [26], and ligaments [27]. By removing proteoglycans and the intercellular matrix between collagen fibrils, the T likely facilitated the entry of its S diluent into fibrils causing them to swell, increasing the fibril diameter as seen in SAXS analyses of the samples. In TEM sections, the fibrils also appeared larger with considerable variation in interfibrillar spacing, very irregular packing and poor short-range order, all consistent with the lack of transparency noted in the transparency testing.

Storage in S only resulted in a significant increase in collagen fibril diameter and fibril diameter distribution. This was probably because endothelial cells on its inner surface maintain the cornea in a slightly dehydrated state [28, 29]. As S is relatively hypotonic, with the loss of endothelial and epithelial cells we noted, water likely moved into the corneas increasing the hydration status of the fibrils, causing them to swell and have larger fibril diameters and distributions as we found in our SAXS and TEM. The resultant mild corneal odema would have interfered with the optimal regular spacing and size of fibrils required for normal transparency [30, 31] and explains their loss of clarity in the transparency test.

## Limitations

Our SAXS analysis only provided an average picture of the collagen layers across the cornea. Recent micro-focus X-ray studies have shown the layers are not uniform with collagen fibril size and direction varying by depth [32]. Access to this technique would have provided us with more precise data on collagen changes at various depths in the cornea. We describe only the general changes readers can expect in parameters measured by SAXS; detailing statistically significant changes requires data on large numbers of samples of each species and age-related changes in collagen would need consideration. Studies are indicated to determine if changes we observed in normal corneas also occur in diseased corneas.

## Supplementary Information

**Additional file 1**: ** Figure S1.** Images for subjective assessment of the transparency of sheep and cats’ preserved corneas on a 2 mm grid.

## Data Availability

The datasets used and/or analyzed during the current study are available from the corresponding author on reasonable request.

## Abbreviations

SAXS: Small angle X-ray scattering
TEM: Transmission electron microscopy
GUI: Graphical user interface

## References

[1] Brodsky B, Persikov AV (2005). Molecular structure of the collagen triple helix. Adv Protein Chem.

[2] Yang W, Sherman VR, Gludovatz B, Schaible E, Stewart P, Ritchie RO (2015). On the tear resistance of skin. Nat Commun.

[3] Meek K, Boote C (2004). The organization of collagen in the corneal stroma. Exp Eye Res.

[4] Meek KM, Knupp C (2015). Corneal structure and transparency. Progr Retinal Eye Res.

[5] Knupp C, Pinali C, Lewis PN, Parfitt GJ, Young RD, Meek KM (2009). The architecture of the cornea and structural basis of its transparency. Adv Protein Chem Struct Biol.

[6] Glatter O, Kratky O (1982). Small angle X-ray scattering. Acta Ploym.

[7] Boote C, Kamma-Lorger CS, Hayes S, Harris J, Burghammer M, Hiller J (2011). Quantification of collagen organization in the peripheral human cornea at micron-scale resolution. Biophys J.

[8] de la Cuesta FB, Wenger MPE, Bean RJ, Bozec L, Horton MA, Robinson IK (2009). Coherent X-ray diffraction from collagenous soft tissues. Proc Natl Acad Sci USA.

[9] McCally R, Farrell R (1982). Structural implications of small-angle light scattering from cornea. Exp Eye Res.

[10] Quantock A, Boote C, Young RD, Hayes S, Tanioka H, Kawasaki S (2007). Small-angle fibre diffraction studies of corneal matrix structure: a depth-profiled investigation of the human eye-bank cornea. J Appl Crystallogr.

[11] Fratzl P, Daxer A (1993). Structural transformation of collagen fibrils in corneal stroma during drying. An X-ray scattering study. Biophys J.

[12] Meek K, Boote C (2009). The use of X-ray scattering techniques to quantify the orientation and distribution of collagen in the corneal stroma. Progr Retinal Eye Res.

[13] Gyi T, Meek KM, Elliott G (1988). Collagen interfibrillar distances in corneal stroma using synchrotron X-ray diffraction: a species study. Int J Biol Macromol.

[14] Bolfa P, Kelly S, Wells H, Sizeland H, Scott E, Kirby N (2018). Tropical keratopathy (Florida spots) in cats. Vet Pathol.

[15] Akhtar S (2012). Effect of processing methods for transmission electron microscopy on corneal collagen fibrils diameter and spacing. Microsc Res Techniq.

[16] Fullwood N, Meek K (1993). A synchrotron X-ray study of the changes occurring in the corneal stroma during processing for electron microscopy. J Microsc.

[17] Turunen MJ, Khayyeri H, Guizar-Sicairos M, Isaksson H (2017). Effects of tissue fixation and dehydration on tendon collagen nanostructure. J Struct Biol.

[18] Hayes S, Boote C, Lewis J, Sheppard J, Abahussin M, Quantock AJ (2007). Comparative study of fibrillar collagen arrangement in the corneas of primates and other mammals. Anat Rec.

[19] Boote C, Hayes S, Abahussin M, Meek K (2006). Mapping collagen organization in the human cornea: left and right eyes are structurally distinct. Invest Ophthalmol Vis Sci.

[20] Bancroft JD, Gamble M (1977). Theory and practice of histological techniques. J Clin Pathol.

[21] Ilavsky J, Jemian P (2009). Irena: tool suite for modeling and analysis of small-angle scattering. J Appl Crystallogr.

[22] Craig A, Robertson J, Parry D (1986). Preservation of corneal collagen fibril structure using low-temperature procedures for electron microscopy. J Ultrastruct Mol Struct Res.

[23] Meek K, Chapman J (1985). Glutaraldehyde-induced changes in the axially projected fine structure of collagen fibrils. J Mol Biol.

[24] Jester J (2008). Corneal crystallins and the development of cellular transparency. Semin Cell Dev Biol.

[25] Grauss R, Hazekamp M, Oppenhuizen F, van Munsteren C, Gittenberger-de Groot A, DeRuiter M (2005). Histological evaluation of decellularised porcine aortic valves: matrix changes due to different decellularisation methods. Eur J Cardiothorac Surg.

[26] Dahl S, Koh J, Prabhakar V, Niklason L (2003). Decellularized native and engineered arterial scaffolds for transplantation. Cell Transplant.

[27] Cartmell J, Dunn M (2004). Development of cell-seeded patellar tendon allografts for anterior cruciate ligament reconstruction. Tissue Eng.

[28] Qazi Y, Wong G, Monson B, Stringham J, Ambati B (2010). Corneal transparency: genesis, maintenance and dysfunction. Brain Res Bull.

[29] Maurice D (1972). The location of the fluid pump in the cornea. J Physiol.

[30] Mazzotta C, Balestrazzi A, Traversi C, Baiocchi S, Caporossi T, Tommasi C (2007). Treatment of progressive keratoconus by riboflavin-UVA-induced cross-linking of corneal collagen: ultrastructural analysis by Heidelberg Retinal Tomograph II in vivo confocal microscopy in humans. Cornea.

[31] Meek K, Dennis S, Khan S (2003). Changes in the refractive index of the stroma and its extrafibrillar matrix when the cornea swells. Biophys J.

[32] Abass A, Hayes S, White N, Sorensen T, Meek KM (2015). Transverse depth-dependent changes in corneal collagen lamellar orientation and distribution. J R Soc Interface.

