# Supplementary material for: Pilot study on the effects of preservatives on corneal collagen parameters measured by small angle X-ray scattering analysis
Source: BMC Res Notes. 2021 Feb 27;14:78. doi: 10.1186/s13104-021-05494-y (PMC7913446; doi:10.1186/s13104-021-05494-y)
Supplement: Supplementary file 1 — Additional file 1: Figure S1. Images for subjective assessment of the transparency of sheep and cats’ preserved corneas on a 2 mm grid. [file 13104_2021_5494_MOESM1_ESM.docx]

**Additional file 1**

**
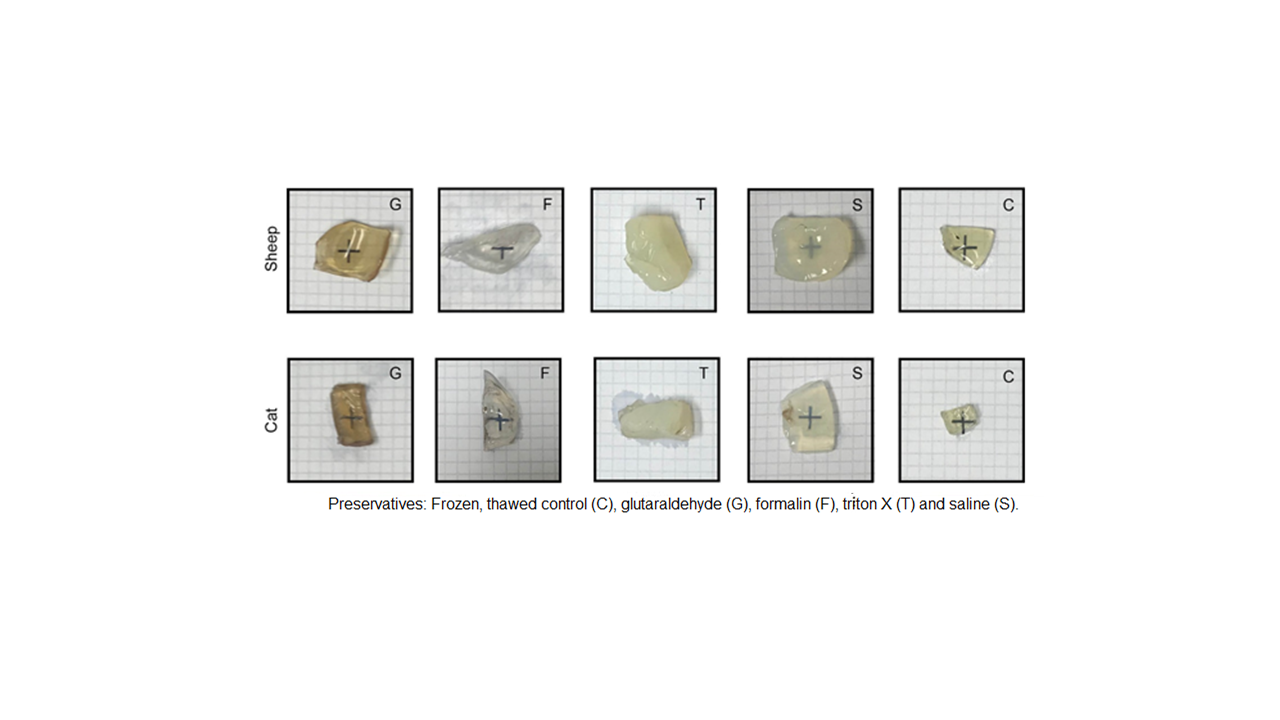
**

**Figure S1.**  Images for subjective assessment of the transparency of sheep and cats’ preserved corneas on a 2 mm grid.
